# Study of the Thermochemical Surface Treatment Effect on the Phase Precipitation and Degradation Behaviour of DSS and SDSS

**DOI:** 10.3390/ma13010165

**Published:** 2020-01-01

**Authors:** Alisiya Biserova-Tahchieva, Jose Maria Cabrera, Nuria Llorca-Isern

**Affiliations:** 1 CPCM, Dept. Ciència de Materials i Química Física, Facultat de Química, Universitat de Barcelona, E-08028 Barcelona, Spain; nullorca@ub.edu; 2 PROCOMAME, Dept. Ciència dels Materials i Enginyeria Metal·lúrgica, EEBE, Universitat Politècnica de Catalunya, E-08019 Barcelona, Spain; jose.maria.cabrera@upc.edu; 3 Institute of Metallurgical Research, Universidad Michoacana de San Nicolás de Hidalgo UMSNH, Av. Francisco Múgica s/n, CU, Morelia 58230, Mexico

**Keywords:** corrosion, duplex stainless steel, plasma ion carburizing, superduplex stainless steel, secondary phases, thermal treatments

## Abstract

In this study, the effect of a plasma ion carburizing process to duplex and superduplex stainless steels (DSS and SDSS), at 925 °C for a long time, as thermochemical process influencing the microstructural evolution is presented. The objective is to analyse the diffusion elements’ influence on the precipitation of secondary phases after additional short thermal treatment. A comparison among the different treatments was performed after the resulting microstructures were analysed by Field Emission—Scanning Electron Microscope. Precipitation of secondary phases—sigma (σ), chi (χ), nitrides and carbides—seemed to occur during the treatments in a similar way for both steels (DSS and SDSS), although they showed a different morphology and precipitation mode. General corrosion behaviour of untreated and treated samples was investigated by potentiodynamic tests in order to prove their corrosion resistance. It was found that an improvement of the surface protection after the plasma carburizing process occurred.

## 1. Introduction

Duplex stainless steels (DSS) are nowadays one of the most widely used groups of steels and are extensively applied in the petrochemical industry for example as oil and gas containers, mainly due to their mechanical properties as well as their corrosion resistance. The combination of austenite and ferrite phases in almost equal percentage exerts a powerful effect on both mechanical and degradation properties of the duplex and superduplex stainless steels [1,2,3,4,5,6]. However, the performance of DSSs can be limited in particular conditions. For instance, DSS corrosion behaviour can be adversely affected under high temperature processes such as welding. As a result, microstructural rearrangement can significantly decrease corrosion resistance and some mechanical properties relevant for specific applications [2,7,8,9,10,11].

Additionally, they are sometimes unsatisfying expected wear and friction properties. This drawback has recently enhanced increasingly rapid advances in the field of surface modification technologies applied to this family of steels. There are different procedures to improve stainless steels surface properties. With the aim of increased hardness and wear resistance, thermochemical diffusion such as nitriding, carburizing, carbonitriding or nitrocarburizing is among the most industrially carried out processes. Generally, this is considered a poor practice because of the temperature range, from 500 to 950 °C, that results in precipitation of chromium nitrides (Cr_2_N) or carbides (M_23_C_6_, M_7_C_3_) known as sensitization. Consequently, the improvement in surface hardness because of these secondary phases is frequently accompanied by a loss in corrosion resistance when the surface treatment is carried out at high temperatures.

To affront this issue, plasma-based treatments were considered advantageous over gaseous processes as removing the natural passive layer of the stainless steels facilitates the inward diffusion of carbon/nitrogen. Moreover, plasma processes enable high temperatures (T_p_ > 900 °C) which circumvent the formation of unwanted nitrides or carbides associated with the depletion of Cr or low temperatures (T_p_ < 450 °C) where metastable expanded austenite (γ_2_) is formed because of the supersaturated nitrogen or carbon solid solution that increases to a large extent the hardness of the surface [12,13,14,15].

Plasma carburizing appears to be a promising alternative for such hardness and wear resistance improvement. It is a thermochemical process consisting of the diffusion of carbon at a sufficient temperature through the surface layer of usually low-carbon steels and high-chromium steels [16], among others. Carburized layers are then fixed between 0.8 and 1.0 wt% carbon. At high temperature, carbon shows its maximum solubility at the stable equilibrium phase of austenite. After that, hardening of the components is achieved by rapid cooling and tempering or cooling down slowly and then reheating to the austenitizing temperature to maintain the very hard surface. The carbon gradient content below the surface determines the hardness of the surface layer, which is usually wear resistant [16]. Plasma ion carburizing is considered an advantageous alternative procedure also due to the oxygen-free atmosphere operation. It is basically a vacuum process using glow-discharge technology to introduce active soluble carbon to the steel surface for its subsequent diffusion, producing greater carburized case depth than gas carburizing [16]. This method has increased its interest in many applications, partly due to its faster carburizing rates, which are associated with a faster rate of carbon saturation, and to the effectively avoided steps in the dissociation process that produce active soluble carbon. Active carbon can be formed directly by the ionizing effect of the plasma. Hence, carbon saturation and carbon diffusion can be easier controlled to follow a greater uniform carburized layer. Moreover, oxides are avoided in the grain boundaries, thus fatigue properties of components are improved through this process [16]. It is generally a temperature and time-dependence diffusion process that follows the square root of time relation [17]. Thus, low temperature and high temperature plasma carburizing results will also depend on the processing time.

On the other hand, despite the higher temperatures allowed to carry out the plasma carburizing treatment, the control of high temperature and geometry of samples have limited the plasma-based techniques, especially for industrial applications. Thus, many authors in the literature are focused on investigating the low temperature plasma carburizing process and its implication in the hardness improvement of families of steels in correlation to the corrosion behaviour [14,15,16,17,18,19]. Most of the studies have emphasized the mechanical and tribocorrosion properties of low carbon steels after carburizing processes at low temperature. However, few studies have investigated the evolution of the microstructure after the plasma carburizing process at a high temperature and for a long duration. It is very well known that during the exposure to high temperatures (600–1000 °C), microstructural changes occur and may cause serious problems. For instance, precipitation of secondary phases such as sigma (σ) and chi (χ) as well as carbides and nitrides, as mentioned previously, may deteriorate mechanical and corrosion resistance of the material. In addition, since alloying elements and, especially carbon diffusion, influence the microstructure evolution of stainless steels, investigation in high temperature plasma carburizing effects on duplex stainless steels, seems to be necessary.

The aim of this project, therefore, is to evaluate the influence of the thermochemical treatment of plasma carburizing at a high temperature over a long period on the microstructure evolution of duplex and superduplex stainless steel and their general corrosion behaviour after this treatment.

## 2. Materials and Methods

**Figure 1:**
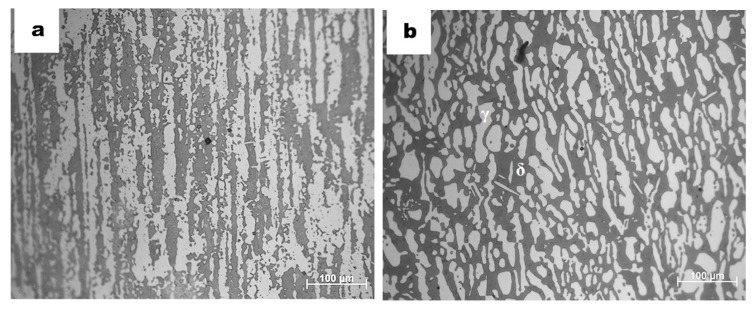
Optical micrographs of DSS (**a**) and SDSS (**b**), after annealing process at 1080 °C for 30 min. Homogenous microstructure of relatively equal distribution of austenite and ferrite phases is shown after being etched by modified Murakami chemical reagent [22].

Samples of duplex stainless steel (UNS S2205) of 2-mm-thick hot-rolled plate and superduplex stainless steel (UNS S2750) of 10-mm-thick hot rolled tub were used in this investigation. Chemical composition in weight percent is shown in Table 1. Previously, all samples were isothermally annealed at 1080 °C for 30 min followed by water quenching in order to obtain a homogenous two-phase microstructure, where austenite phase (γ-fcc) is distributed in the matrix of the delta ferrite phase (δ-bcc) without secondary phases precipitated (Figure 1). Afterwards, all specimens were polished to a mirror finish with successive SiC papers, diamond pastes and colloidal silica.

**Figure 2:**
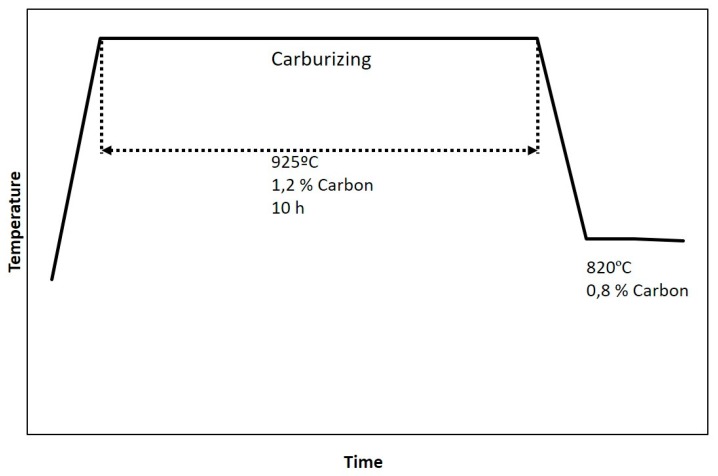
Schematic representation of the carburizing cycle holding at 925 °C for 10 h, quenched to 820 °C, followed by cooling in air.

Thermochemical surface treatment of carburizing was carried out through the plasma carburizing process in carbon atmosphere at 925 °C over 10 h. The scheme of the process is shown in Figure 2. It is worth mentioning the followed cooling in air, as no hardness increase was meant to be achieved by this carburizing process, but phase precipitation. In order to analyse any further precipitation after the carburizing process, short holding time post-thermal treatments were carried out at 830 ± 2 °C to carburize samples followed by water quenching. These conditions of thermal treatment were selected in order to compare the results to previous precipitation research [20].

In parallel to this treatment, isothermal aging at the same temperature and during the same time—925 °C, 10 h—was carried out after homogenization of the samples in order to compare the secondary phases to those after the carburizing process.

High resolution scanning electron microscope (FE-SEM) JEOL J-7100F (JEOL Ltd, Tokyo, Japan) with a coupled Robinson BSE detector and energy dispersive X-ray spectroscopy (EDS) was used to characterize the carburized layer and to determine semi-quantitatively the phase’s chemical composition.

Potentiodynamic polarization tests were performed following ASTM [21] standard by using AUTOLAB potentiometer (Metrohm Autolab, Utrecht, The Netherlands). The electrochemical cell was a three-electrode system with a Pt mesh electrode as a counter against the sample as a working electrode with ~2 cm^2^ area exposed and a reference electrode of silver–silver chloride (Ag/AgCl 3 M). All tests were performed at room temperature using 3.5 wt% NaCl solution and the potential magnitude is given with respect to the reference electrode denoted as V_SCE_. Samples were first conditioned to the stabilized potential for 3 h, then the open circuit potential (OCP) was measured for 1 h. At least three polarization tests were conducted to ensure the reproducibility of the results. The potential range was scanned from −0.5 to near 0.5 V_SCE_ at a scan rate of 1 mV·s^−1^.

## 3. Results and Discussion

In order to better understand the formation of the secondary phase’s precipitation mechanism and consequently elucidate a better control for this deleterious effect, microstructure evolution was studied following carbon diffusion through the treatments carried out to DSS and SDSS samples. This evolution was also correlated to the corrosion resistance behaviour showed by the samples at the different treatment conditions.

### 3.1. Microstructural Evolution After Carburizing Process

**Figure 3:**
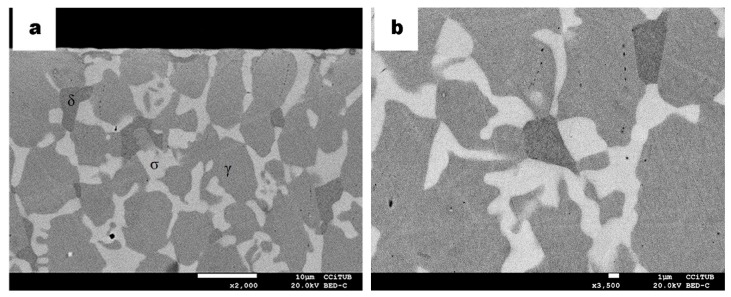
BSE micrograph of DSS carburized samples, (**a**) showing γ (grey phase) and ferrite δ (greyer phase) as well as sigma σ (bright phase). (**b**) Higher magnification of a region away from the surface; some dark points are identified as chromium carbides (dark phase).

**Figure 4:**
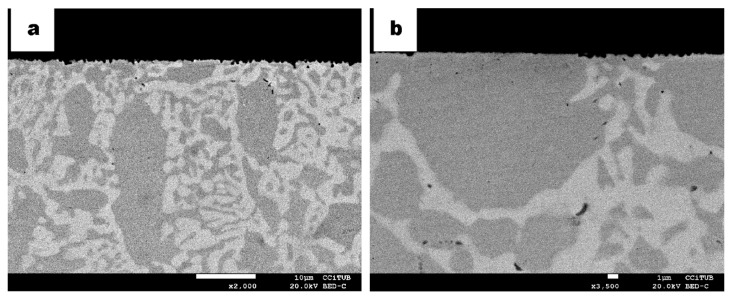
BSE micrograph of SDSS carburized samples (**a**) indicating γ (grey phase), sigma phase σ (bright phase) and chromium carbides (dark phase); (**b**) higher magnification of an area at the surface.

In Figure 3, micrographs of the DSS 2205 and SDSS 2750 plasma carburized samples for 10 h at 925 °C are shown. Precipitation of the secondary sigma phase (bright phase indicated as σ in Figure 3) occurs in an obvious abundance. Similar precipitation of the sigma phase but in less abundance was previously detected in duplex and superduplex stainless steel samples thermally treated at 830 °C for less than 5 min without any kind of surface thermochemical processes carried out [22]. Precipitation of heterogeneously and intragranularly dark spots are also assigned as carbides. The carbon supplied to the substrate combines with iron and chromium largely in the form of carbides. The formation of these secondary phases confirms the diffusion saturation of C that reaches the solubility limit in the austenite–ferrite microstructure [14]. Such carbides are mainly of the type M_23_C_6_ (when M stands for Fe/Cr) and are also reported in other studies [13,23]. On the other hand, DSS sample does not show oxides when compared with slight oxides that appeared in the surface of SDSS sample as shown in Figure 4a, b. Internal oxidation is typical in gas carburizing processes due to the penetration of oxygen, where the atmosphere contain partial pressures of CO, CO_2_ and H_2_O leading to some internal oxidation of the samples [24,25]. However, in plasma carburizing, such an issue can be discarded, and no intragranular oxidation is observed as expected.

Figure 4 micrographs show the microstructure obtained for the SDSS sample. A different morphology of the brighter phase among the austenite phase is observed. Such a phase was identified as a sigma phase. A similar transformation has been identified in previous studies as eutectoid transformation of ferrite to sigma phase and secondary austenite (δ → σ + γ_2_) [26,27]. The ferrite phase in this SDSS sample has been completely transformed compared to the microstructure observed in Figure 3a, b of the DSS sample, in which only incipient transformation is detected. Carbides were identified also near the surface that are attributed to the supersaturation of carbon after its diffusion by the plasma process.

### 3.2. Microstructural Evolution After Carburizing Process and Short Aging Treatment

**Figure 5:**
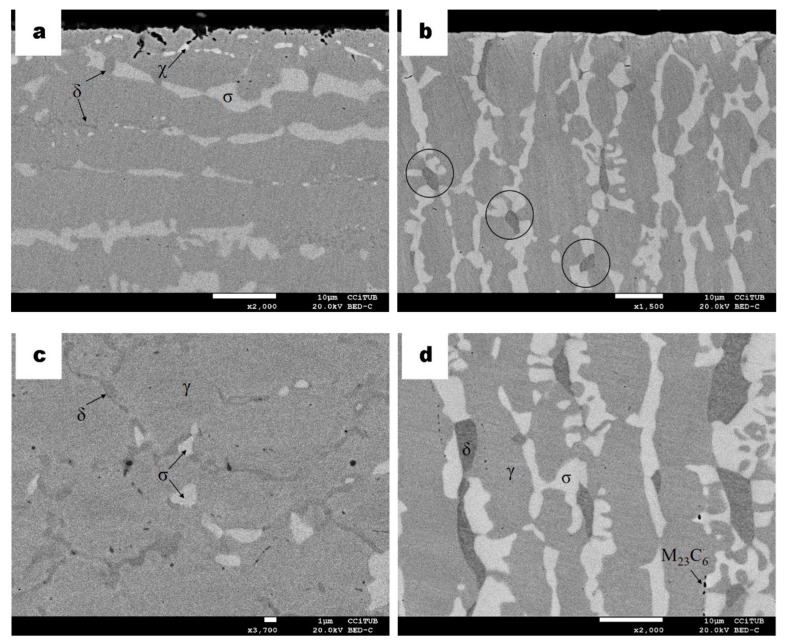
BSE micrographs of cross-section DSS specimens after 10 h plasma carburizing at 925 °C and thermal treatment at 830 °C for a short time. (**a**) Microstructure of the surface indicating ferrite δ (greyer phase) as well as sigma σ (bright phase) and chi χ (brightest phase). (**b**) Longitudinal cross section of the microstructure indication butterfly-like precipitation with black circles. (**c**) Higher magnification of central zone indicating austenite γ (grey phase), δ and σ. (**d**) Higher magnification of (b) indicating chromium carbides (black phase).

In view of the microstructures and the absence of secondary chi phase after the large thermochemical cycle, samples were then aged for around 3 min at 830 °C in order to see the effect of the short thermal treatment as seen in previous samples after the same aging [22]. The cross-sectional microstructural images of the carburized DSS sample with an additional thermal cycle are shown in Figure 5. The micrographs show almost similar microstructures as the ones in Figure 3. Nevertheless, observing the transversal microstructure of DSS in Figure 5a, a brighter phase than sigma elongated in the rolling direction near the surface of the sample is observed. Its characterization by semiquantitative EDS with higher content in Mo than sigma phase confirms the identification as chi-phase (indicated as χ in Figure 5a).

The precipitation of the chi phase use to happen in a previous stage of sigma phase [2,22,27,28,29,30]. At higher temperature or thermal treatment duration, the chi phase usually converts into the sigma phase. Hence, as seen in the micrographs, the sigma phase is appearing in a greater amount, partly due to the high presence of chromium as well. Most of this secondary phase is distributed in the rolling direction of the sample, involving the ferrite phase. It is also possible the precipitation of both sigma and chi phases simultaneously at a particular temperature and some residual chi phase can also be detected while the sigma phase progresses in its precipitation [3,5,22,30,31,32,33,34,35,36]; this is why both phases are observed in Figure 5a.

The particular morphology of the sigma phase, which is similar to the same butterfly shape reported by Nilsson et al. [27], is also observed in Figure 5b (marked with circles). Its nucleation occurs at ferrite/ferrite/austenite triple point. Compared to the precipitation shown in Figure 3a,b, after the aging process, the sigma phase shows more elongated morphology within the thinner austenite phase along it. Carbides still appear randomly in the core and case of the carburized sample after the additional aging treatment as observed in the cross section of the DSS sample (Figure 5c). However, longitudinally, as seen in the micrograph in Figure 5b, carbides have been less detected, at least at the surface. At a different region inside the sample, the displacement of such carbides can be seen from the γ/σ boundary. Such displacement could be due to several microstructural distortions such as grain boundary movement, phase boundary displacement or dislocation movements (Figure 5d).

The thermodynamics and kinetics of SDSS and DSS differ. For instance, the kinetics of nucleation and growth of secondary phases is faster for the former than the latter. Since the intermetallic phases are enriched in Cr and Mo, the higher content of these elements in UNS S32750 (SDSS) leads to a greater formation of such phases than in the UNS S32205 (DSS).

**Figure 6:**
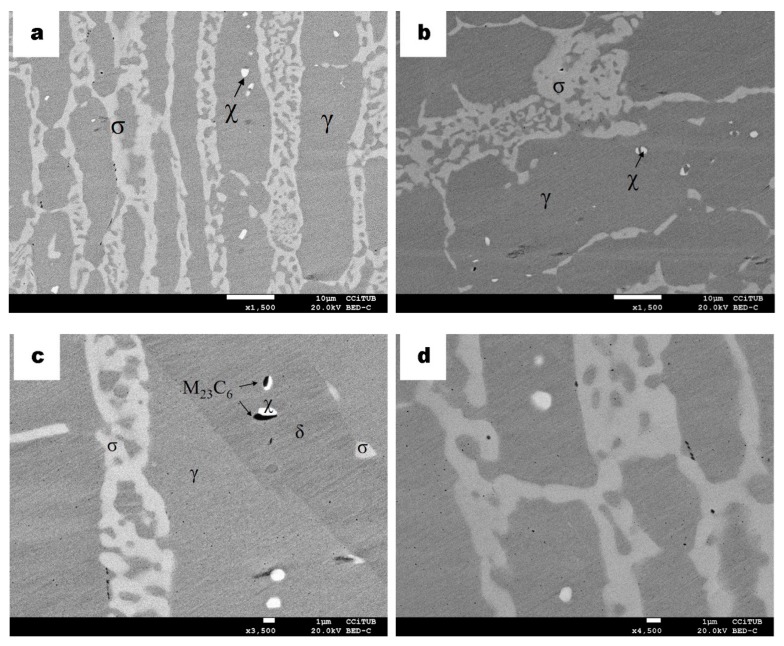
BSE micrographs of SDSS specimen cross-sections after 10 h plasma carburizing at 925 °C and thermal treatment at 830 °C for short time. (**a**) Details of the microstructure indicating austenite (γ), ferrite (δ), sigma (σ), chi χ (brightest phase) and chromium carbides (dark spots). (**b**–**d**) are different zones more in deep of the sample, showing the nucleation site of the chi-phase, which is M_23_C_6_.

As can be seen in the microstructure of the micrographs in Figure 6, chi phase precipitated in the austenite (Figure 6a,b,d) and in the δ ferrite phase (Figure 6c) in the SDSS sample after the same carburizing and annealing process (830 °C for 3 min) as processed with the DSS sample. Sigma phase precipitation is predominant and elongated in the ferrite phase due to its higher diffusion rate and the higher content of Cr and Mo in this ferrite phase [37]. Moreover, M_23_C_6_ has also been found to nucleate in the ferrite phase and seems to be an initial site of nucleation for the chi-phase (Figure 6a–c). This mechanism differs from what was found without the plasma carburizing for the same alloys. The main role of carbon is the formation of carbides, M_23_C_6_ and Cr_7_C_3_. In the absence of nitrides or in detrimental amount compared to them, these carbides accomplish the nucleation site for χ phase.

### 3.3. Microstructural Evolution After Isothermal Aging Treatment

An isothermal aging treatment at the same temperature as carburizing, 925 °C for 10 h, was carried out in order to observe the precipitation of the secondary phases in the samples and then these were compared to the ones precipitated after the carburizing process.

**Figure 7:**
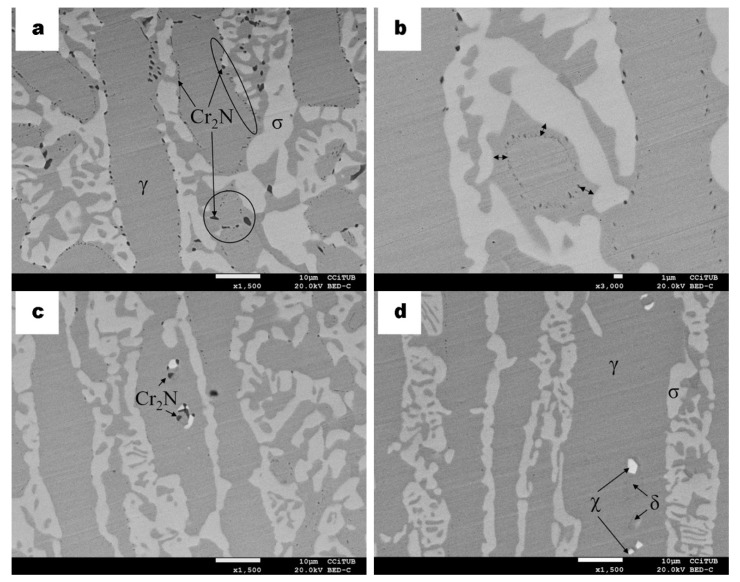
BSE micrographs of SDSS specimen cross-sections after isothermal aging for 10 h at 925 °C. (**a**) Microstructure of the sample near the surface, indicating austenite (γ), ferrite (δ), sigma (σ) and chromium nitrides (dark spots). (**b**) Higher magnification of a zone near the surface indicating with arrows the displacement of the nitrides from the γ/σ boundary, forming a circle. (**c**,**d**) show the microstructure away from the surface of the sample indicating chi-phase nucleation.

As seen in the micrographs of SDSS samples in Figure 7, precipitation of the sigma phase is almost equal to the sigma detected in SDSS after carburizing (Figure 6). However, micrographs in Figure 7a,b show an increase of dark spots near the surface of the sample. Such small little dark spots were also detected in the samples after carburizing and were assigned to the precipitation of carbides. In contrast, dark spots encircled in Figure 7a were analyzed and correspond to the precipitation of nitrides. According to the literature, almost all nitrides found in SDSS are assigned to Cr_2_N [1,38]. A displacement of these nitrides from the γ/σ boundary can also be seen, which has been denoted previously, due to several microstructural distortions and as the sigma phase takes place. Thus, the identified nitrides form a circle between the boundaries of γ/σ (Figure 7b). Besides, as seen in Figure 6c, in Figure 7c it is observed that the secondary chi phase also has an initial point for nucleation, which is the already formed nitride at the boundary of γ/δ. Additionally, as seen in Figure 7d, the chi-phase appears directly on the boundary between γ/δ.

From this aging treatment, it has been seen that in the absence of carbon concentration, in comparison with the carburizing process, nitrides precipitate preferably near the surface of the SDSS samples instead of carbides and as a barrier and/or at γ/σ boundaries.

**Figure 8:**
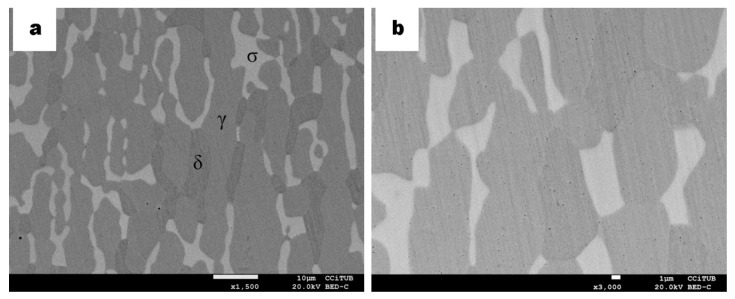
BSE micrographs of cross-section DSS specimens after isothermal aging for 10 h at 925 °C. (**a**) Microstructure of the sample indicating ferrite δ (greyer phase) as well as sigma σ (bright phase) and austenite γ (grey phase). (**b**) Higher magnification of central zone. Black spots presented heterogeneously in both micrographs are assigned to chromium carbides.

On the other hand, carbides are the ones predominating in the DSS samples, heterogeneously distributed almost like in the samples after carburizing. The absence of nitrogen, which probably dissolved in the austenite phase, was verified by EDS analysis, which then lead us to confirm that the dark phases were carbides (Figure 8a,b). Moreover, the ferrite phase is still appearing, as in the DSS samples after the carburizing cycle, but there is no visible displacement of carbides as seen in Figure 3b and Figure 5d or at least it has not been detected at this particular point of the sample.

In order to determine if the carburizing process is superficially effective while internal precipitation occurs and does not alter the material on the surface, in the sense of decreasing protection against corrosion, plasma carburized samples were tested by a potentiodynamic polarization before and after annealing treatment. Thus, general corrosion behaviour has been analysed as follows.

### 3.4. Potentiodynamic Polarization Tests

Potentiodynamic polarization test is a very useful experiment to determine general corrosion behaviour. It is an excellent method to identify parameters such as the corrosion potential (E_corr_) and the corrosion current density (j_corr_) by analysing the obtained polarization graphs. This test helps to compare qualitatively the protection for different systems as well.

**Figure 9:**
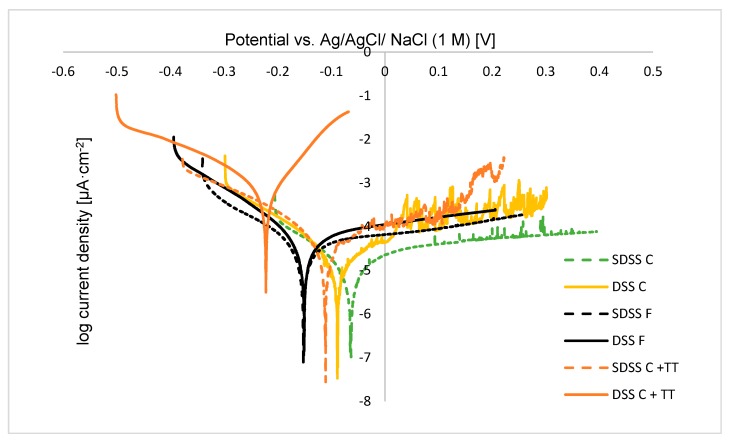
Potentiodynamic curves of DSS and SDSS samples as received (F), after the plasma carburizing process (C), after plasma carburizing and additional thermal treatment at 830 °C for a short time (C + TT).

Figure 9 shows the anodic polarization curves or potentiodynamic curves measured in a solution of 3.5%wt NaCl for untreated (DSS F and SDSS F), plasma carburized samples (DSS C and SDSS C) and plasma carburized samples with additional thermal treatment at 830 °C (DSS C + TT and SDSS C + TT). Almost all the tested specimens showed good corrosion resistance as expected. Untreated samples (DSS F and SDSS F) present quite similar values of E_corr_ due to their similar corrosion behaviour. When compared to the untreated samples, the plasma carburizing process of both samples (DSS C and SDSS C) carried out at 925 °C slightly improved the corrosion behaviour even though internal precipitation of the sigma phase was detected in a significant amount. This is an important issue in order to apply surface modification thermal treatments. This beneficial protection can also be seen by the low measured current densities as presented in Table 2. The summarized values (E_corr_ and i_corr_) extracted from the potentiodynamic polarization curves were calculated by carrying out the cathodic-anodic Tafel slope. Moreover, the corrosion potential of samples without plasma carburizing, DSS F (−0.149 ± 0.004 V) and SDSS F (−0.143 ± 0.014 V) is higher than that of the plasma carburized samples DSS C (−0.089 ± 0.022 V) and SDSS C (−0.064 ± 0.010 V). The obtained information for the untreated samples (DSS F and SDSS F) can be comparable to previous studies [39,40]. Knowing that as more positive the corrosion potential and lower the current density, as better the corrosion behaviour, there is an improvement of the surface protection in the corrosion behaviour after the plasma carburizing process. On the other hand, E_corr_ becomes more negative (worse corrosion resistance) for samples of DSS C + TT (−0.261 ± 0.033 V) in comparison with the untreated and plasma carburized samples, indicating that corrosion behaviour slightly decreases. BSE micrographs of the plasma carburized samples showed a slightly different aspect when additionally treated at 830 °C after the plasma carburizing process. For instance, for the DSS C + TT sample, the precipitation of the chi-phase near the surface seems that affects and it is revealed by the potentiodynamic polarization behaviour, where an increase in the corrosion potential was analysed. As it is a superficial test, the presence of the chi phase in the sub surface of the DSS sample easily provoked a decrease in its corrosion resistance. However, the presence of the chi-phase in the core of the SDSS C + TT, and not on the surface, leads to the fact that the relatively low distribution of carbides in this sample does not harm it from the corrosion properties point of view.

Usually carbides and nitrides are responsible for the decrease in corrosion resistance in such processes at low temperatures [13,25,41]. Herein, due to the extremely low amount of nitrogen, the small dark phases were identified mainly as carbides. These are distributed heterogeneously and have not appeared to be harmful for the surface properties of the plasma carburized samples.

Polarization curves in Figure 9 and values in Table 2 show the differences between samples and treatments. Experiments have evidenced that plasma carburized samples have not shown passivation potential within the potential range from −0.5 to 0.5 V_SCE_ at a scan rate of 1 mV·s^−1^. There are no visible passivation regions corresponding to abrupt increments in current density as can be seen in the curves shown in Figure 9.

It is relevant to discuss the oscillatory region observed between ~0.0 and ~0.3 V for the carburized samples. Such behaviour is observed previously [42,43] and is typical of metals immersed in chloride solutions. However, the DSS C + TT sample does not present such behaviour. The sharp fluctuations in current density are defined as the metastable pit growth that initiates below the pitting potential, but they cannot propagate for an indefinite period and, therefore, repassivate fairly quickly [44,45,46,47]. In addition, as mentioned before, no pitting potential is reached.

Regarding the plasma carburizing at lower temperatures, carbon presents lower diffusivity, and consequently, low solubility in the ferrite phase (0.0025%) [48]. Therefore, the adsorbed carbon atoms on the surface do not have enough mobility to diffuse into the substrate, a saturation of the carbon on the surface is then caused and cementite is precipitated after fast cooling. However, at higher temperature and cooling in the air as in this study, carbon presents diffusion depth enhancement [17]. The increased solubility of the ferrite matrix for carbon and the high content of Cr in the δ phase, during a large time of diffusion, should lead to the precipitation of chromium carbides; however, at the same time, ferrite transforms into sigma phase (σ). The precipitation of chi-phase secondary phases is enhanced after an additional aging treatment at 830 °C because of the temperature range. Moreover, the sigma phase is a predominant phase when compared to chromium carbides. A higher amount of chromium is maintained in the solid solution, and the precipitation of carbides is mostly heterogeneous and not near the surface [13,49,50]. Therefore, potentiodynamic polarization test results have not shown a decrease in the corrosion behaviour of the samples tested superficially.

## 4. Conclusions

Plasma-carburizing process, as a thermochemical surface modification process, and its influence on the microstructure evolution of DSS (US32205) and SDSS (US32507) materials have been carried out. Precipitation of secondary phases occurred at a temperature of 925 °C during the large period and after normal cooling. A sigma phase after the plasma cycle has been identified extensively in both duplex and superduplex samples. However, higher diffusion of elements in the superduplex material was observed as greater transformation was clearly detected. Almost all ferrite phase in the SDSS were transformed into a sigma phase, resulting in an elongated morphology along the austenitic phase in the rolling direction. Additional thermal treatment at 830 °C for a shorter time to the DSS carburized samples revealed an intermetallic chi-phase, richer in Mo (wt%), near the surface, which consequently promoted the decrease in corrosion resistance. On the other hand, a chi-phase in the SDSS was found in the inner microstructure. Chromium carbides were the generally preferable nucleation sites for this phase. These carbides have been observed homogenously in both samples and have not shown to have a significant influence on the corrosion decrease. A displacement of some carbides was detected from the γ/σ boundaries, which is attributed to different microstructural distortions.

A comparison between the carburizing process at a high temperature during a large period of time (normal cooling afterwards) and isothermal aging treatment at the same temperature and time (quenching afterwards) showed similar precipitated phases. However, in the absence of carbon, nitrides tend to precipitate; these and the ferrite/austenite boundaries are the nucleation sites for the chi-phase.

In conclusion, the plasma-carburizing procedure at a relatively high temperature was found to be an effective protection for the surface, from the corrosion behaviour point of view. Corrosion potentials were almost equal for both DSS and SDSS samples, and although precipitation occurred interiorly, the surface of the sample, being thermochemically protected, was not altered.

## Figures and Tables

**Table 1:** Chemical composition of duplex (S32205) and super duplex (S32750) stainless steels (Fe balanced).

Sample Identification	Chemical Composition wt%									
UNS	C	Si	Mn	P	S	Cr	Ni	Mo	N	Cu
S32205	0.015	0.40	1.50	0.018	0.001	22.49	5.77	3.21	0.18	0.18
S32750	0.018	0.26	0.84	0.019	0.001	25.08	6.88	3.82	0.29	0.17

**Table 2:** Experimental parameters of corrosion potential and current density of the corresponding samples. Data are expressed as mean ± standard deviation (n = 3).

	SDSS C	DSS C	SDSS C + TT	DSS C + TT	SDSS F	DSS F
E corr [V]	−0.064 ± 0.010	−0.089 ± 0.022	−0.111 ± 0.057	−0.261 ± 0.033	−0.143 ± 0.014	−0.149 ± 0.004
jcorr [μA/cm^2^]	0.007 ± 0.002	0.016 ± 0.009	0.028 ± 0.049	0.594 ± 0.034	0.026 ± 0.009	0.021 ± 0.002
